# Artificial Intelligence for Biomedical Diagnostics: Diagnostic Accuracy and Reliability of Multimodal Large Language Models in Electrocardiogram Interpretation

**DOI:** 10.3390/life16040681

**Published:** 2026-04-16

**Authors:** Henrik Stelling, Armin Kraus, Gerrit Grieb, David Breidung, Ibrahim Güler

**Affiliations:** 1 Practices for Nuclear Medicine, Rubensstraße 125, 12157 Berlin, Germany; henrikstelling@googlemail.com; 2 Department of Health Management, Friedrich-Alexander-Universität Erlangen-Nürnberg (FAU), Lange Gasse 20, 90403 Nuremberg, Germany; 3 Department of Plastic, Aesthetic and Hand Surgery, Otto-von-Guericke University, 39120 Magdeburg, Germany; armin.kraus@med.ovgu.de (A.K.); ibrahim.gueler@med.ovgu.de (I.G.); 4 Department of Plastic Surgery and Hand Surgery, Gemeinschaftskrankenhaus Havelhöhe, Kladower Damm 221, 14089 Berlin, Germany; gerritgrieb@gmx.de; 5 Department of Plastic Surgery and Hand Surgery, RWTH Aachen University, Pauwelsstrasse 30, 52074 Aachen, Germany; 6 Department of Health, University of Witten/Herdecke, 58455 Witten, Germany; david.breidung@usz.ch; 7 Department of Plastic Surgery and Hand Surgery, University Hospital Zurich, Rämistrasse 100, 8091 Zurich, Switzerland

**Keywords:** electrocardiography, electrocardiogram interpretation, cardiovascular diagnostics, diagnostic accuracy, biomedical signal analysis, multimodal large language models, artificial intelligence, cardiac rhythm analysis, clinical electrocardiography, cardiovascular physiology

## Abstract

The electrocardiogram (ECG) is a central tool in cardiovascular diagnostics, yet interpretation requires expertise and remains subject to variability. Multimodal large language models (MLLMs) have shown emerging capabilities in medical image analysis, but their performance in ECG interpretation remains insufficiently characterized. This study evaluated the diagnostic accuracy and inter-run reliability of five MLLMs across ECG interpretation tasks. Thirteen standard 12-lead ECGs were presented to five models (ChatGPT-5.3, Gemini 3.1 Pro, Claude Opus 4.6, Grok 4.1, and ERNIE 5.0) across five independent runs per case, yielding 2275 task-level assessments. Six categorical interpretation tasks (rhythm, electrical axis, PR/P-wave morphology, QRS duration, ST/T-wave morphology, and QTc interval) were compared with expert-consensus ground truth, while heart rate estimation was evaluated using mean absolute error (MAE). Overall categorical accuracy ranged from 52.3% to 64.9%. QRS duration classification achieved the highest accuracy (66.2–90.8%), whereas ST/T-wave assessment showed the lowest performance (20.0–41.5%). Heart rate MAE ranged from 14.8 to 46.7 bpm. A dissociation between diagnostic accuracy and inter-run reliability was observed across models. These findings indicate that current MLLMs do not achieve clinically reliable ECG interpretation performance and highlight the importance of assessing diagnostic accuracy and inter-run reliability when evaluating artificial intelligence systems in biomedical diagnostics.

## 1. Introduction

The 12-lead electrocardiogram (ECG) is among the most widely performed diagnostic tests in clinical medicine, with millions of recordings obtained annually worldwide. Accurate ECG interpretation is essential for the timely diagnosis of arrhythmias, ischemia, conduction abnormalities, and structural heart disease. However, ECG interpretation is a skill-dependent task that requires extensive training, and diagnostic accuracy varies considerably among clinicians of different specialization levels. Even among cardiologists, inter-observer agreement for specific ECG findings can be limited, raising questions about the reliability of human ECG interpretation in routine clinical practice [1,2,3,4]. These well-documented limitations have motivated the development of computer-assisted ECG analysis over several decades, beginning with rule-based algorithms and progressing toward increasingly sophisticated machine learning approaches.

Artificial intelligence (AI) approaches to ECG analysis have a long history, ranging from rule-based algorithms embedded in electrocardiograph devices, which remain the clinical standard for automated ECG interpretation, to convolutional neural network (CNN)-based classifiers trained on large-scale annotated databases that have demonstrated cardiologist-level performance for specific diagnostic tasks [5]. More recently, multimodal large language models (MLLMs) have emerged as a distinct paradigm: these general-purpose models process both text and images without dedicated training for electrocardiogram interpretation, offering potential as flexible diagnostic assistants. Unlike conventional ECG algorithms that output predefined classifications, MLLMs can engage in open-ended interpretation of ECG images, a capability that has attracted growing research interest [6,7]. As these models have become more widely accessible, an emerging body of literature has begun to systematically evaluate their diagnostic performance, revealing both partial capabilities and important limitations. Several recent studies have evaluated the performance of MLLMs in ECG interpretation across heterogeneous experimental settings, including multiple-choice formats, open-ended diagnosis, and binary versus multiclass classification tasks. Zhu et al. reported that GPT-4V achieved up to 83.9% accuracy on ECG multiple-choice questions but performed poorly on open-ended diagnostic tasks [8]. Bocz et al. found that ChatGPT-4 and Gemini achieved only 31% and 26% accuracy, respectively, on 130 clinical ECGs compared to expert consensus [9]. Çamkıran et al. observed accuracies of 58–63% on 107 ECGs using GPT-based models, substantially below the 92.5% accuracy of cardiologists [10]. Engelstein et al. reported 83% accuracy for binary classification but only 41% for multiclass ECG diagnosis using GPT-4o [11]. Collectively, these findings suggest that while MLLMs demonstrate partial capabilities in ECG interpretation, their performance remains inconsistent, task-dependent, and below clinical-grade reliability. A more detailed comparison of the present study with these prior works is provided in the discussion (Section 4.4).

A common limitation of existing evaluations is the reliance on single-run study designs, in which each model is queried only once per case. This approach cannot capture the stochastic nature of MLLM outputs, which may vary across repeated queries due to temperature-dependent sampling and other sources of non-determinism [12,13]. Therefore, accuracy estimates derived from single runs may overestimate or underestimate true model capability. The need for multi-run evaluation protocols has been highlighted in recent methodological work on MLLM benchmarking [14,15]. A second limitation is the frequent reduction in ECG assessment to a single task, typically rhythm classification, whereas clinical ECG interpretation requires systematic evaluation of rate, rhythm, axis, intervals, and waveform morphology [16].

The present study addresses both gaps by evaluating five MLLMs on seven ECG interpretation tasks across five independent runs per model. This design permits not only estimation of diagnostic accuracy per task but also assessment of inter-run reliability using Fleiss’ κ computed on actual response categories. By jointly reporting accuracy and consistency, this study aims to provide a more comprehensive characterization of current MLLM capabilities for ECG interpretation than single-run, single-task evaluations allow. Specifically, the contributions of this work are as follows: (1) a multi-model comparison of five MLLMs across seven distinct ECG interpretation tasks using a structured evaluation framework; (2) an assessment of inter-run reliability using Fleiss’ κ to quantify the relationship between diagnostic accuracy and output consistency; and (3) an analysis of task-dependent performance patterns in relation to the known difficulty hierarchy of human ECG interpretation.

## 2. Materials and Methods

### 2.1. Study Design and Objectives

This cross-sectional exploratory benchmarking study evaluated the diagnostic accuracy and inter-run reliability of five MLLMs on the interpretation of standard 12-lead ECGs. Each model was queried five times per ECG in independent sessions, yielding 325 model responses corresponding to 2275 task-level assessments across 13 ECGs and seven interpretation tasks (six categorical and one continuous). The outcomes were diagnostic accuracy and inter-run reliability (consistency) across tasks.

### 2.2. Dataset and Case Selection

Thirteen standard 12-lead ECGs were selected. Each ECG comprised six limb leads (I, II, III, aVR, aVL, aVF) and six precordial leads (V1–V6). The dataset included both normal and pathological ECG findings, encompassing a range of heart rates (50–160 beats per minute [bpm]; mean 104.6 bpm), rhythms (11 regular, 2 irregular), electrical axes (10 normal, 2 left axis deviation, 1 right axis deviation), QRS morphologies (11 narrow, 2 wide), and ST/T-wave abnormalities (2 T-wave inversions, 1 ST elevation, 1 ST depression). Ground truth labels were based on clinically pre-interpreted ECGs and were subsequently reviewed by the study authors, all of whom have clinical experience in ECG interpretation. Labels for all seven tasks were independently assessed and, where necessary, harmonized through discussion to ensure consistency across the dataset.

### 2.3. Models Under Evaluation

Five MLLMs with image processing capabilities were evaluated: ChatGPT-5.3 (OpenAI, San Francisco, CA, USA), Gemini 3.1 Pro (Google DeepMind, Mountain View, CA, USA), Claude Opus 4.6 (Anthropic, San Francisco, CA, USA), Grok 4.1 (xAI, San Francisco, CA, USA), and ERNIE 5.0 (Baidu, Beijing, China). All models were accessed through their official web interfaces using default settings. No application programming interface (API) parameters were modified, and no system prompts beyond the standardized evaluation prompt were applied. Specifically, temperature and other decoding parameters were not user-adjustable through the web interfaces, and no modifications to default sampling behavior were possible. The identical standardized evaluation prompt was used for all models across all runs, with the complete prompt structure, including all task definitions and constrained response categories, described in Section 2.4.

### 2.4. Prompting Strategy and Inference Protocol

A zero-shot prompting approach was used [17]. Each ECG image was presented to each model with an identical, standardized prompt requesting structured assessment of seven parameters: heart rate (numeric estimate in bpm), rhythm (regular or irregular), electrical axis (normal, left axis deviation, or right), PR interval/P-wave morphology (normal, not visible, or polymorphic P-waves), QRS duration (narrow or wide), ST-segment/T-wave morphology (normal, elevated, slight lateral ST depression, T-wave inverted, or not assessable), and QTc interval (normal, prolonged QT interval, J wave visible after the QRS, or not assessable). The prompt constrained each response to predefined categorical options to enable standardized comparison across models. Each model was queried five times per ECG in separate, independent sessions to prevent carry-over effects. A new conversation was initiated for each run.

### 2.5. Outcome Definitions

Seven ECG interpretation tasks were evaluated (six categorical and one continuous), and the outcomes were diagnostic accuracy and inter-run reliability across tasks. The six categorical tasks were: rhythm classification (binary: regular vs. irregular), electrical axis determination (3 categories), PR interval/P-wave assessment (3 categories), QRS duration classification (binary: narrow vs. wide), ST-segment/T-wave assessment (5 categories), and QTc interval evaluation (4 categories). The continuous task was heart rate estimation (bpm). Table 1 summarizes the task definitions, response categories, and ground truth distributions.

### 2.6. Statistical Analysis

Diagnostic accuracy for categorical tasks was computed as the proportion of correct responses across all five runs per model (N = 65 responses per model–task combination). Results are reported as accuracy (%) per task and overall. 95% confidence intervals were calculated on task-level responses using the Wilson method and should be interpreted descriptively, reflecting the precision of observed proportions within the evaluated sample rather than supporting population-level inference. For binary classification tasks (rhythm and QRS duration), additional diagnostic performance metrics (sensitivity, specificity, positive predictive value [PPV], negative predictive value [NPV], and balanced accuracy) were calculated using majority vote predictions per ECG (N = 13), whereby the most frequent response across five runs was taken as the model’s prediction for each case. Balanced accuracy was defined as the arithmetic mean of sensitivity and specificity, providing a prevalence-independent performance measure. These metrics are reported in Supplementary Table S1 and should be interpreted descriptively given the small number of cases per class. Per-class diagnostic metrics were not reported for multi-class tasks (3–5 categories) due to the small number of cases per category, which would yield unstable and potentially misleading estimates.

For heart rate estimation, mean absolute error (MAE) was calculated for each run as the mean of the absolute differences between model estimates and ground truth values across the 13 ECGs. Results are reported as the mean MAE across five runs ± standard deviation (SD) of the five run-level MAEs.

Inter-run reliability was assessed using Fleiss’ κ, computed on the actual response categories produced by each model [18]. For each model-task combination, κ was calculated treating the 13 ECGs as items and the five independent runs as raters. This approach captures whether a model assigns the same label to the same ECG across repeated queries, irrespective of correctness (hereafter referred to as consistency). κ values were interpreted according to Landis and Koch: less than 0.20 as slight, 0.21–0.40 as fair, 0.41–0.60 as moderate, 0.61–0.80 as substantial, and 0.81–1.00 as almost perfect agreement [19]. All predefined response options, including “not assessable,” were treated as valid categories for both accuracy and κ calculations. Results are reported per task and as a mean across tasks.

Majority-class baselines were computed for all categorical tasks to contextualize accuracy relative to the expected performance of a naive classifier that always predicts the most frequent ground truth label. An overall baseline (75.6%) was calculated as the arithmetic mean of the six task-specific majority-class accuracies. Because this aggregate combines tasks of differing complexity and number of categories, it should be interpreted as a descriptive benchmark rather than a formal statistical threshold. Task-specific baselines ranged from 61.5% (ST/T-wave) to 84.6% (rhythm, QRS) for the dominant category. The high baselines for rhythm (84.6%) and QRS (84.6%) reflect the binary nature of these tasks combined with a dataset in which normal findings predominate (11 of 13 ECGs), which is representative of clinical ECG populations where the majority of recordings show regular rhythm and narrow QRS complexes.

Where high accuracy coincided with low κ values, the κ paradox was considered as a potential explanation. This statistical artifact arises when prevalence imbalance causes high marginal agreement, compressing the denominator of the κ statistic and yielding misleadingly low values despite adequate agreement [20].

The statistical framework of this study was deliberately chosen to match its design and scale. With 13 independent ECG cases evaluated across five repeated runs per model, the study yields 65 observations per model–task combination; however, these observations are not independent, as the repeated-measures structure violates the independence assumption underlying standard frequentist tests such as McNemar, chi-square, or unadjusted ANOVA. Inferential between-model testing was therefore not performed, as the resulting *p*-values would rest on an assumption the design does not satisfy and would convey a level of certainty that the data cannot support.

Confidence interval widths depend on the level at which the data are considered. When calculated on the 65 task-level responses, the half-width is approximately ±12 percentage points around an observed accuracy of 50%, narrowing to approximately ±7 percentage points at the extremes. When conservatively interpreted at the level of the 13 independent ECG cases, the corresponding uncertainty increases to approximately ±24 percentage points around 50%, reflecting the limited number of independent observations. This distinction is inherent to the repeated-measures design and underscores the need to interpret all estimates as descriptive. Inter-run reliability is reported separately as Fleiss’ κ, which is the appropriate target estimation for the multi-run design.

All statistical analyses were performed using Python 3.12 with standard statistical and data visualization libraries [21].

## 3. Results

### 3.1. Overview of Diagnostic Accuracy

**Figure 1:**
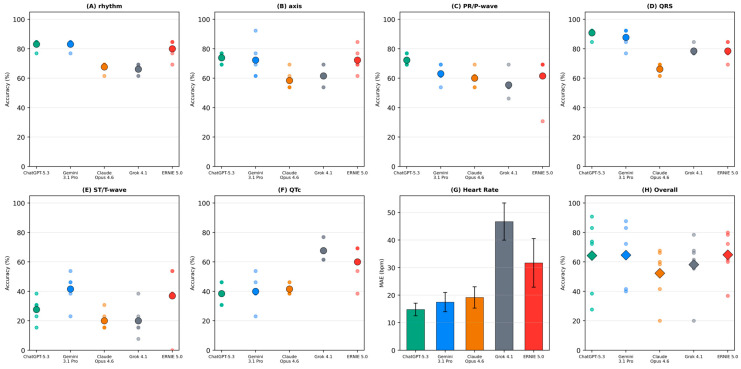
Multi-panel overview of model performance across ECG interpretation tasks. Each small translucent dot represents accuracy from one of five independent inference runs per model; large colored dots indicate mean accuracy across runs. Panel (**A**): rhythm classification; (**B**): axis determination; (**C**): PR interval/P-wave morphology; (**D**): QRS duration; (**E**): ST-segment/T-wave abnormalities; (**F**): QTc interval assessment; (**G**): heart rate estimation error (bars = MAE, whiskers = SD); (**H**): overall categorical accuracy (diamonds = mean across six categorical tasks, small dots = run-level accuracies). Panels (**A**–**F**,**H**) use accuracy (%); Panel (**G**) uses MAE in beats per minute. Model colors: ChatGPT-5.3 (green), Gemini 3.1 Pro (blue), Claude Opus 4.6 (orange), Grok 4.1 (gray), ERNIE 5.0 (red). MAE = Mean Absolute Error; SD = Standard Deviation.

Overall categorical accuracy across all six tasks ranged from 52.3% (Claude Opus 4.6) to 64.9% (ERNIE 5.0), with ChatGPT-5.3 (64.4%), Gemini 3.1 Pro (64.6%), and Grok 4.1 (58.2%) in between. The overall majority-class baseline across all tasks was 75.6%. No model exceeded this overall majority-class baseline (Table 2, Figure 1H).

### 3.2. Task-Level Accuracy

A clear task-dependent hierarchy of diagnostic accuracy was observed (Table 2, Figure 1A–F). QRS duration classification yielded the highest accuracy across models (66.2–90.8%), and ChatGPT-5.3 (90.8%) and Gemini 3.1 Pro (87.7%) were the only models to exceed the majority-class baseline for any task (baseline 84.6%). Rhythm classification (66.2–83.1%), electrical axis determination (58.5–73.8%), and PR/P-wave assessment (55.4–72.3%) showed intermediate accuracy, with all models remaining below their respective baselines. ST/T-wave morphology produced the lowest accuracy (20.0–41.5%), with all models performing well below the 61.5% baseline. QTc interval evaluation showed a similar pattern (38.5–67.7% vs. baseline 69.2%).

### 3.3. Heart Rate Estimation

Heart rate MAE ranged from 14.8 bpm (ChatGPT-5.3) to 46.7 bpm (Grok 4.1) (Table 3, Figure 1G). Three models achieved MAEs below 20 bpm (ChatGPT-5.3: 14.8 ± 2.3; Gemini 3.1 Pro: 17.5 ± 3.5; Claude Opus 4.6: 19.2 ± 3.9), while ERNIE 5.0 (31.7 ± 8.8) and Grok 4.1 (46.7 ± 6.7) showed larger deviations. Inter-run variability in MAE, reflected in the SD, ranged from 2.3 to 8.8 bpm.

### 3.4. Inter-Run Reliability

Fleiss’ κ values varied substantially across models and tasks (Table 4). Claude Opus 4.6 showed the highest mean κ (0.727), with values ranging from moderate (axis: κ = 0.548) to almost perfect (rhythm: κ = 0.938). ChatGPT-5.3 achieved the second-highest mean κ (0.605), with substantial to almost perfect agreement on four of six tasks but only slight agreement for rhythm (κ = 0.126). Gemini 3.1 Pro demonstrated moderate mean reliability (κ = 0.513). Grok 4.1 (mean κ = 0.198) and ERNIE 5.0 (mean κ = 0.030) showed predominantly slight agreement, with ERNIE 5.0 producing negative κ values on two tasks (PR/P-wave: κ = −0.078; QTc: κ = −0.116), indicating agreement worse than chance.

### 3.5. Accuracy–Consistency Dissociation

**Figure 2:**
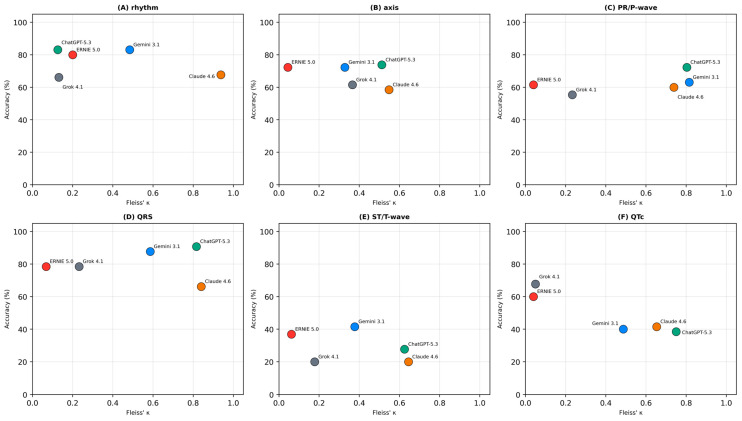
Task-specific accuracy–consistency profiles. Models are plotted according to mean categorical accuracy (*y*-axis) and inter-run reliability measured by Fleiss’ κ (*x*-axis). Panel (**A**): rhythm classification; (**B**): axis determination; (**C**): PR interval/P-wave morphology; (**D**): QRS duration; (**E**): ST-segment/T-wave abnormalities; (**F**): QTc interval assessment. Models achieving high accuracy with low κ demonstrate the accuracy–consistency dissociation: correct answers on average but inconsistent across repeated runs.

A dissociation between accuracy and inter-run consistency was observed across models (Figure 2). The clearest contrast was between ERNIE 5.0 and Claude Opus 4.6: ERNIE 5.0 achieved the highest overall categorical accuracy (64.9%) but the lowest mean κ (0.030), while Claude Opus 4.6 showed the reverse pattern (accuracy 52.3%, mean κ = 0.727). ChatGPT-5.3 combined above-average accuracy (64.4%) with above-average consistency (mean κ = 0.605), representing the most balanced profile. Gemini 3.1 Pro achieved similar accuracy (64.6%) but lower consistency (mean κ = 0.513). Grok 4.1 showed below-average performance on both dimensions (accuracy 58.2%, mean κ = 0.198).

At the task level, simpler morphological assessments (QRS duration) permitted both higher accuracy and higher consistency, while complex waveform interpretations (ST/T-wave, QTc) combined lower accuracy with variable reliability.

## 4. Discussion

### 4.1. Task-Dependent Performance Hierarchy

The present study reveals a clear task-dependent performance hierarchy. QRS duration classification, a binary distinction between narrow and wide complexes with clearly delineated morphological boundaries, yielded the highest accuracy, with two models exceeding the task-specific majority-class baseline for an individual task. In contrast, ST-segment and T-wave assessment, which requires differentiation among different morphological categories involving subtle amplitude and slope changes across multiple leads, produced the lowest accuracy, with all models performing below the corresponding baseline. This gradient from simple binary features to complex multi-class waveform assessments parallels the known difficulty hierarchy of human ECG interpretation: QRS morphology is among the earliest competencies acquired during ECG training, whereas ST/T-wave analysis involves pattern recognition across spatial lead relationships and remains error-prone even among experienced clinicians [3,4]. A comparable pattern has been observed for GPT-4o in ECG interpretation, where performance was high for simple image recognition (100%) but substantially lower for classification tasks, particularly in zero-shot binary classification (53%) and multiclass diagnosis (41%), suggesting that performance declines with increasing task complexity, particularly in the absence of task-specific guidance [11].

Several factors may explain why ST/T-wave assessment proved substantially more difficult than QRS duration classification. First, ST- and T-wave abnormalities involve subtle morphological deviations that require sub-millimeter amplitude judgment relative to the isoelectric baseline, integration across multiple leads to identify reciprocal changes, and discrimination between visually similar categories such as concave versus convex elevation or biphasic versus inverted T-waves. QRS duration, in contrast, reduces to a single width measurement against a binary threshold (120 ms) that is recognizable in any individual lead and therefore represents a more discrete and quantifiable feature. Second, it is plausible that the vision encoders of current general-purpose MLLMs, which compress input images into a limited set of visual tokens, attenuate exactly the low-contrast, pixel-level features on which ST/T-wave recognition depends, while coarser morphological features such as QRS width are more likely to be preserved under this compression. This would be consistent with the observation that general-purpose multimodal models appear to rely on abstracted visual–textual representations rather than true waveform-level analysis.

The poor performance on ST/T-wave and QTc assessment is clinically significant. ST-segment deviations are the primary electrocardiographic markers of myocardial ischemia and infarction, and QTc prolongation is associated with increased risk of life-threatening arrhythmias [22,23]. The observation that all five models performed below the majority-class baseline on both tasks indicates that current models do not achieve sufficient performance for the ECG parameters that carry the highest diagnostic weight in acute care settings.

Heart rate estimation showed a similar pattern of model differentiation. The three best-performing models achieved MAEs of 14.8 to 19.2 bpm, which translates to clinically relevant misclassifications at the tachycardia (>100 bpm) and bradycardia (<60 bpm) thresholds. The two remaining models showed MAEs exceeding 30 bpm, rendering their rate estimates unreliable for clinical application.

### 4.2. Accuracy–Consistency Dissociation

A central finding of this study is the dissociation between diagnostic accuracy and inter-run consistency, highlighting a notable limitation of single-run evaluation designs that are commonly used in MLLM benchmarking. The model with the highest overall categorical accuracy simultaneously produced the lowest mean Fleiss’ κ across tasks, including negative values on two parameters. Conversely, the most consistent model, with the highest mean κ, achieved the lowest overall accuracy. These findings are consistent with prior work demonstrating a dissociation between diagnostic accuracy and model consistency, indicating that models may produce reproducible yet systematically incorrect outputs [14]. This pattern is also reflected in controlled multi-run evaluations: Günay and Yiğit repeatedly administered identical ECGs (with accompanying clinical context) to GPT-4o and DeepSeek and reported that DeepSeek achieved lower diagnostic accuracy but substantially higher inter-run agreement (Fleiss’ κ = 0.71) compared to GPT-4o (Fleiss’ κ = 0.47), consistent with the accuracy–consistency dissociation observed across models in the present study [24].

The practical implications of this dissociation are considerable. In a clinical decision support scenario, a model with high accuracy but low consistency would provide unreliable outputs, potentially generating correct interpretations on initial deployment but failing on subsequent consultations for identical cases. A model with low accuracy but high consistency, while predictably wrong, would at least permit systematic identification and correction of its error patterns. Neither profile is clinically acceptable, but the distinction is relevant for understanding model behavior and guiding future development [12,13].

From a clinical safety perspective, inter-run reliability represents an independent dimension of model performance beyond diagnostic accuracy, and reproducibility is a prerequisite for validation, auditability, and regulatory qualification of diagnostic systems. A model whose output varies across identical inputs cannot be reliably benchmarked against a reference standard or integrated into established quality-assurance workflows, independently of its average accuracy. The accuracy–reliability dissociation observed here therefore implies that single-query use of current MLLMs in clinical decision support is not defensible on reliability grounds alone, and that any prospective deployment in a screening or triage context would require explicit mitigation strategies such as multi-run majority voting, quantification of output uncertainty across repeated inferences, or mandatory human confirmation of each individual output. The multi-run design represents a key methodological contribution of this study, as it enables assessment of inter-run variability, a property not captured in single-run evaluations and directly relevant to the reliability of real-world model deployment.

ChatGPT-5.3, which combined above-average accuracy with above-average consistency, exhibited the most balanced profile, suggesting that accuracy and consistency are not inherently mutually exclusive. This aligns with recent work emphasizing the need to account for inter-run variability when evaluating LLM performance [15].

### 4.3. The Kappa Paradox in MLLM Evaluation

The κ paradox, described by Feinstein and Cicchetti [20], may be relevant to several model–task combinations in the present study, most prominently for rhythm classification, where models achieving accuracy near the majority-class baseline produced low κ values. This pattern is consistent with response distributions skewed toward the dominant category, yielding high marginal homogeneity that compresses the κ denominator, and may partly reflect a statistical artifact of the interaction between response bias and category prevalence rather than true model instability.

The κ paradox is well recognized in inter-rater reliability assessment but has received limited attention in the MLLM evaluation literature. Given that many medical classification tasks involve imbalanced class distributions, future studies should routinely report both κ values and response distributions to enable correct interpretation of reliability estimates [25].

### 4.4. Comparison with Prior Literature

To situate the present findings within the rapidly expanding body of work on MLLMs for ECG interpretation, Table 5 summarizes selected prior studies alongside the current evaluation. The studies differ substantially in dataset size, input format, task design, model generation, and evaluation protocol, which limits direct numerical comparison. The table is therefore intended to support a structured qualitative comparison rather than a quantitative meta-analytic synthesis.

Across the prior literature, reported accuracy values for image-based ECG interpretation by general-purpose MLLMs vary widely, typically ranging from approximately 19% to 66%, depending strongly on task framing. Studies that constrain the response space, for example through multiple-choice formats or binary endpoints, tend to yield higher headline accuracies than studies requiring open free-text diagnosis. Within free-text designs, accuracies on heterogeneous ECG cohorts typically fall between 25% and 60%, with substantial variation between model families and between routine and more challenging cases [8,9,10,24,26,27,28,29]. The accuracy range observed in the present study (52.3–64.9% overall categorical accuracy across five models) is broadly consistent with this body of work and likely reflects the combination of more recent model generations, the use of constrained categorical response options for each interpretation task, and a multi-task structure that includes both relatively tractable parameters (such as rhythm and QRS duration) and known points of weakness for current MLLMs (such as ST/T-wave morphology and QTc estimation).

Two design features distinguish the present study from most prior work. First, only a small number of studies have so far assessed inter-run reliability through repeated administration of identical inputs to the same model. Zhu et al. used three repetitions per item in a multiple-choice setting [8]; Engelstein et al. evaluated multiple prompt formulations rather than strict repetitions and additionally performed repeated runs of the best-performing setup to assess robustness [11]; and Günay and Yiğit administered identical ECG cases with accompanying clinical context thirteen times to GPT-4o and DeepSeek, reporting Fleiss’ κ values of 0.47 and 0.71, respectively, although the design does not isolate image-based reasoning [24]. The five-run design used here is therefore aligned with this small but growing methodological strand and complements the accuracy estimates with task-level inter-run agreement. Second, in contrast to prior work that has typically reported overall correctness or a single binary endpoint, the present study evaluates seven structured interpretation parameters in parallel, which permits a task-resolved view of where accuracy and reliability dissociate.

Several broader observations from the literature are reinforced by the present findings.

- First, dedicated ECG-specific architectures and instruction-tuned ECG models continue to outperform general-purpose MLLMs by a wide margin on directly comparable tasks, underscoring that current general-purpose MLLMs may not yet represent a substitute for domain-specific systems [26,29,30].
- Second, accuracy is highly sensitive to task framing, prompt structure, and the availability of clinical context, which limits the external validity of any single accuracy estimate, including those reported here [28].
- Third, headline accuracy values can mask substantial inter-run variability, which supports the central methodological argument of the present study that single-run benchmarking is insufficient for diagnostic evaluation of MLLMs and that reliability should be reported alongside accuracy [24].

The present study contributes to this emerging literature by providing a multi-model, multi-task, multi-run evaluation of the most recent MLLM generation, and by quantifying the accuracy–reliability dissociation in a structured manner.

**Table 5:** Comparison of the present study with selected prior evaluations of multimodal large language models for ECG interpretation.

Study	N Unique ECG Images	Models Evaluated	Runs	Reported Accuracy (and κ Where Available)
Present study	13	ChatGPT-5.3, Gemini 3.1 Pro, Claude Opus 4.6, Grok 4.1, ERNIE 5.0	5	Overall categorical accuracy 52.3–64.9% across models; mean Fleiss κ 0.03–0.73 across models
Günay 2024 [27]	40	GPT-4, GPT-4o, Gemini Advanced	12	Median ~51% (GPT-4) to ~67.5% (GPT-4o); Fleiss κ 0.27–0.51
Günay & Yiğit 2026 [24]	40	GPT-4o, DeepSeek R1	13	Median correct answers 22/40 (DeepSeek) vs. 27/40 (GPT-4o); Fleiss κ 0.71/0.47
Zhu 2024 [8]	62	GPT-4V	3 attempts per item	MCQ-based; 53.2% strict (3/3 correct) vs. 83.9% lenient (≥1/3 correct)
Engelstein 2025 [11]	80	GPT-4o, Gemini 2.0 Flash	Mixed (3 prompt formats; 5 runs of best setup)	Zero-shot binary 53–63%; few-shot binary 83%; 6-class 41%
Çamkıran 2025 [10]	107	GPT-ECGReader, GPT-ECGAnalyzer, GPT-ECGInterpreter	1	57.9–62.6% across the three GPT variants
Bocz 2025 [9]	130	GPT-4, Gemini	1	25.6% (Gemini) to 31.2% (GPT-4)
Zeljković 2025 [28]	150	GPT-4	1 (×2 conditions)	19% (without context) vs. 45% (with clinical context)
Seki 2025 [31]	928	GPT-4V, Gemini Pro Vision	1	No single accuracy metric reported; qualitative evaluation only
Lee 2025 [26]	928	GPT-4o, Gemini 2.5 Pro	1	29.6% (Gemini 2.5 Pro) to 66.0% (GPT-4o)
Yang 2025 [29]	n.a. (signal-based; PTB-XL)	ECG-LM (purpose-built multimodal LLM)	n.a.	Domain-specific model; metrics not directly comparable to general-purpose MLLMs
Liu 2026 [30]	ECGBench (multi-dataset)	GPT-4o, GPT-4o mini, Gemini 1.5 Pro, Claude 3.5 Sonnet, and open-source MLLMs (LLaVA-OneVision, DeepSeek-VL, MiniCPM-V)	1	Relative performance reported; domain-tuned PULSE outperforms general-purpose MLLMs by 21–33% in average accuracy

Studies are ordered by the number of unique ECG images (ascending). The present study is highlighted. Reported performance metrics are extracted from each publication and correspond to the primary endpoint defined in the respective study. These metrics include, but are not limited to, accuracy, median correct answers, task-specific scores, and qualitative or semi-quantitative evaluations. Direct comparison across studies is limited due to substantial heterogeneity in study design, including differences in task formulation (e.g., multiple-choice, binary classification, multi-class classification, free-text generation), input modality (signal vs. image), dataset composition, prompting strategies, response constraints, model versions, and evaluation protocols. “Runs” refer to the number of repeated model inferences using identical inputs where reported. n.a. = not applicable.

### 4.5. Clinical Implications and Future Directions

Within the scope of this exploratory evaluation, the findings do not support the use of current MLLMs for independent ECG interpretation in clinical practice. No model exceeded the overall majority-class baseline for categorical accuracy, and the diagnostically most demanding tasks showed the weakest performance. While these observations are limited by the sample size and should be confirmed in larger studies, they suggest that general-purpose MLLMs, when applied to clinical tasks, require rigorous task-specific validation before deployment. This aligns with broader evidence that AI systems in medicine must undergo robust validation, including real-world clinical assessment, prior to implementation [32].

However, the partial competence observed for simpler tasks suggests potential utility in structured screening scenarios where model outputs are confirmed by qualified personnel. Such applications would require careful definition of task scope, performance thresholds, and human oversight mechanisms. More promising paths toward clinically viable AI-assisted ECG interpretation may involve domain-specific training on large-scale annotated ECG databases, structured output frameworks, or specialized foundation models such as the approach proposed by Mallick et al. [6,7]. Direct empirical support for this direction is provided by recent comparative work: Lee et al. reported that a dedicated ECG analysis tool achieved 97.0% accuracy on myocardial infarction detection from ECG images, compared with 66.0% for GPT-4o and 29.6% for Gemini 2.5 Pro evaluated on the same dataset [26]. Similarly, instruction-tuned multimodal models specifically adapted for ECG image interpretation have been shown to outperform leading general-purpose MLLMs by 21–33% in average accuracy [30]. Together, these findings suggest that domain adaptation plays a critical role in improving clinically relevant performance beyond what is achieved by general-purpose models alone. Direct comparisons between general-purpose MLLMs and conventional ECG algorithms (e.g., CNN-based classifiers trained on annotated signal data) would be methodologically informative but require carefully controlled experimental designs that account for fundamental differences in input format, training paradigm, and output structure. Similar challenges in comparing distinct deep learning architectures have been highlighted in the context of medical image analysis, for example in comparisons between convolutional neural networks and transformer-based models [33]. Additionally, parameter-efficient fine-tuning techniques such as LoRA and QLoRA may offer a practical pathway for adapting existing foundation models to ECG-specific tasks without requiring full retraining [34,35]. Further promising directions include retrieval-augmented generation (RAG), which has shown promise for grounding model outputs in verified clinical knowledge in other medical domains [36,37], tool-augmented inference, and hybrid pipelines combining visual feature extraction with structured clinical reasoning. Recent advances in AI-driven cardiology, including applications in cardiac electrophysiology [38], suggest that adapting such techniques to ECG interpretation represents a feasible next step.

### 4.6. Limitations

This study has several limitations.

- First, the dataset comprised 13 unique ECGs, yielding 325 model responses and 2275 task-level assessments through the multi-run design. While the total number of responses reflects repeated measurements, the number of independent ECG cases is N = 13. The repeated-measures approach was a deliberate methodological choice: assessing inter-run reliability inherently requires multiple observations per case, which was prioritized over maximizing the number of unique ECGs within a single-run design. The dataset does not represent the full clinical ECG spectrum; specific pathologies such as ST-elevation myocardial infarction, atrioventricular block, or bundle branch block were underrepresented or absent. Accordingly, all between-model comparisons should be interpreted as exploratory and descriptive. A formal post hoc power analysis was not performed, as the study does not test a pre-specified hypothesis, and the repeated-measures structure does not align with standard power calculations based on independent observations. Future studies should employ larger datasets with systematic inclusion of defined pathological entities to permit more granular performance analysis. The statistical implications of the limited number of independent cases are addressed quantitatively in Section 2.6, where the precision of all reported accuracy estimates is communicated through 95% Wilson confidence intervals and where the rationale for the descriptive analytical framework is set out in detail.
- Second, all models were accessed through their official web interfaces using default settings. This approach reflects real-world usage conditions but precludes control over inference parameters such as temperature, which may influence output variability. API-based evaluations with fixed temperature settings could yield different consistency profiles.
- Third, only zero-shot prompting was employed. Few-shot or chain-of-thought prompting strategies might improve performance, and the absence of such comparisons limits generalizability to the specific prompting paradigm used.
- Fourth, all prompts were in English. Model performance may differ for prompts in other languages, and language-specific effects on diagnostic accuracy have been documented for text-based medical queries.
- Fifth, model versions evolve rapidly, and the results reported here are specific to the versions evaluated at the time of data collection (March 2026). Given the continuous updates to these systems, performance characteristics may change over time, underscoring the need for longitudinal re-evaluation.
- Sixth, this study assessed only whether model outputs matched ground truth categories, without evaluating the quality of clinical reasoning or the ability to integrate ECG findings with patient context. Models that arrive at correct answers through flawed reasoning may be less reliable in practice than accuracy metrics suggest. Prior work on MLLM ECG interpretation has highlighted discrepancies between predicted labels and underlying explanations, with models often producing inaccurate descriptions of image features even when the final classification is correct. Consistently, qualitative analyses report that the majority of diagnostic explanations are partially or fully incorrect [26,31].
- Seventh, the ECGs were interpreted in isolation, without accompanying clinical information such as patient age, symptoms, or medication history. In clinical practice, ECG interpretation is always performed in context, and the absence of this information may disadvantage models capable of contextual integration. The magnitude of this effect has been quantified in a recent study: when GPT-4 was provided with clinical context for the same set of ECGs, interpretation accuracy increased from 19% to 45%, with the largest improvement observed in acute coronary syndrome cases (10% vs. 70%) [28].
- Eighth, the constrained response format, while enabling standardized comparison across models, may not fully capture model capabilities for open-ended ECG interpretation. Models may identify abnormalities that do not fit the predefined categories, and this potential was not assessed.
- Ninth, ground truth labels were based on clinically pre-interpreted ECGs and subsequently harmonized by the study authors. While this approach reflects pragmatic study design and is consistent with prior work, residual subjectivity cannot be excluded, particularly given the known variability in human ECG interpretation.
- Tenth, the study did not include a concurrent comparison with human experts interpreting the same ECGs under identical conditions. A direct human–AI comparison on the same dataset would provide important contextualization of model performance and is recommended for future work.
- Eleventh, this study evaluated MLLMs in their default configuration without retrieval-augmented generation or access to external medical knowledge bases. The absence of such mechanisms represents both a limitation of the current evaluation and a motivation for future work.

## 5. Conclusions

This multi-run evaluation of five MLLMs across seven ECG interpretation tasks demonstrates that current models do not yet achieve performance levels sufficient for independent electrocardiogram interpretation. Overall categorical accuracy did not exceed the majority-class baseline, and diagnostically critical tasks, including ST/T-wave and QTc assessment, showed the poorest performance.

A key finding is the consistent dissociation between accuracy and inter-run consistency, indicating that models may appear accurate on average while producing unstable outputs across repeated inferences. This undermines the reliability of single-run evaluations and challenges current benchmarking practices.

These results suggest that conventional single-run assessments may not fully capture the variability of MLLM outputs and can give an incomplete picture of their clinical utility. Multi-run evaluation protocols that jointly assess accuracy and reliability should therefore be prioritized in future benchmarking of AI systems in biomedical diagnostics.

## Figures and Tables

**Table 1:** Task definitions, response categories, and ground truth distribution.

Task	Type	Categories	Ground Truth Distribution	Baseline (%)
Heart rate	Continuous	Numeric (bpm)	Range: 50–160; Mean: 104.6 bpm	n/a
Rhythm	Binary	Regular, irregular	Regular: 11; Irregular: 2	84.6
Electrical axis	3-class	Normal, LAD, right axis deviation	Normal: 10; LAD: 2; Right axis dev.: 1	76.9
PR/P-wave	3-class	Normal, not visible, polymorphic	Normal: 10; Not visible: 2; Poly.: 1	76.9
QRS duration	Binary	Narrow, wide	Narrow: 11; Wide: 2	84.6
ST/T-wave	5-class	Normal, ST elevation, ST depression, T-wave inversion, not assessable	Normal: 8; T-wave inv.: 2; ST elev.: 1; ST dep.: 1; N/A: 1	61.5
QTc interval *	4-class	Normal, prolonged, J wave visible, not assessable	Normal: 9; Prolonged: 2; J wave: 1; N/A: 1	69.2

* The “J wave visible” category was included to capture early repolarization patterns that may affect QTc assessment. Response categories reflect the standardized prompt used for all models and do not represent standard clinical QTc classification schemes. n/a: not applicable.

**Table 2:** Diagnostic accuracy (%) per model and task.

Model	Rhythm	Axis	PR/P-Wave	QRS	ST/T-Wave	QTc	Overall
ChatGPT-5.3	83.1 (72.2–90.3)	73.8 (62.0–83.0)	72.3 (60.4–81.7)	**90.8** (81.3–95.7)	27.7 (18.3–39.6)	38.5 (27.6–50.6)	64.4
Gemini 3.1 Pro	83.1 (72.2–90.3)	72.3 (60.4–81.7)	63.1 (50.9–73.8)	**87.7** (77.5–93.6)	41.5 (30.4–53.7)	40.0 (29.0–52.1)	64.6
Claude Opus 4.6	67.7 (55.6–77.8)	58.5 (46.3–69.6)	60.0 (47.9–71.0)	66.2 (54.0–76.5)	20.0 (12.1–31.3)	41.5 (30.4–53.7)	52.3
Grok 4.1	66.2 (54.0–76.5)	61.5 (49.4–72.4)	55.4 (43.3–66.8)	78.5 (67.0–86.7)	20.0 (12.1–31.3)	67.7 (55.6–77.8)	58.2
ERNIE 5.0	80.0 (68.7–87.9)	72.3 (60.4–81.7)	61.5 (49.4–72.4)	78.5 (67.0–86.7)	36.9 (26.2–49.1)	60.0 (47.9–71.0)	64.9
Baseline	84.6	76.9	76.9	84.6	61.5	69.2	75.6

Values represent the proportion of correct responses across five independent runs (N = 65 responses per cell), with 95% Wilson confidence intervals in parentheses. The majority-class baseline is provided for reference. Bold values indicate performance at or above the majority-class baseline. Detailed diagnostic performance metrics for binary tasks are provided in Supplementary Table S1.

**Table 3:** Heart rate estimation.

Model	MAE (bpm)	SD (bpm)	95% CI
ChatGPT-5.3	14.8	2.3	11.9–17.6
Gemini 3.1 Pro	17.5	3.5	13.9–21.0
Claude Opus 4.6	19.2	3.9	15.7–22.7
Grok 4.1	46.7	6.7	40.4–53.0
ERNIE 5.0	31.7	8.8	25.2–38.2

Mean absolute error (MAE), standard deviation (SD), and 95% confidence interval across five independent runs. The CI was calculated on pooled responses (N = 65) for descriptive purposes.

**Table 4:** Inter-run reliability (Fleiss’ κ) per model and task.

Model	Rhythm	Axis	PR/P-Wave	QRS	ST/T-Wave	QTc	Mean κ
ChatGPT-5.3	0.126	0.512	0.803	0.816	0.625	0.750	0.605
Gemini 3.1 Pro	0.484	0.328	0.815	0.586	0.377	0.487	0.513
Claude Opus 4.6	0.938	0.548	0.739	0.840	0.645	0.653	0.727
Grok 4.1	0.131	0.366	0.233	0.232	0.178	0.050	0.198
ERNIE 5.0	0.200	0.044	−0.078	0.068	0.062	−0.116	0.030

Values represent agreement across five independent runs on actual response categories. Mean κ is the unweighted average across six categorical tasks. Interpretation: <0.20 slight, 0.21–0.40 fair, 0.41–0.60 moderate, 0.61–0.80 substantial, 0.81–1.00 almost perfect.

## Data Availability

The standardized evaluation prompt is provided in the Supplementary Materials. The ECG images cannot be shared due to restrictions on redistribution. Additional data are available from the corresponding author upon request.

## Supplementary Materials

The following supporting information can be downloaded at: https://www.mdpi.com/article/10.3390/life16040681/s1, Table S1: Diagnostic performance metrics for binary classification tasks; Text S1: Standardized evaluation prompt.

## Abbreviations

The following abbreviations are used in this manuscript:

AI	Artificial intelligence
API	Application programming interface
bpm	Beats per minute
CNN	Convolutional neural network
ECG	Electrocardiogram
κ	Kappa coefficient
LAD	Left axis deviation
LoRA	Low-rank adaptation
MAE	Mean absolute error
MLLM	Multimodal large language model
QLoRA	Quantized low-rank adaptation
RAG	Retrieval-augmented generation
SD	Standard deviation
