# Supplementary material for: Artificial Intelligence for Biomedical Diagnostics: Diagnostic Accuracy and Reliability of Multimodal Large Language Models in Electrocardiogram Interpretation
Source: Life (Basel). 2026 Apr 16;16(4):681. doi: 10.3390/life16040681 (PMC13117897; doi:10.3390/life16040681)
Supplement: Supplementary file 1 [file life-16-00681-s001.zip › Prompt.pdf]

Standardized Evaluation Prompt

The following prompt was presented identically to all models at the beginning of each independent evaluation session. Each ECG image was uploaded individually in a new conversation together with this prompt. The prompt was provided in English and remained unchanged across all models and runs.

Analyze the attached 12-lead ECG image. For each of the following seven parameters, provide exactly one response from the options listed in parentheses. Do not include explanations, commentary, or differential diagnoses.

1. Heart rate: Provide a numeric estimate in beats per minute (bpm).

2. Rhythm (regular / irregular)

3. Electrical axis (normal / left axis deviation / right axis deviation)

4. PR interval/P-wave morphology (normal / polymorphic P-waves / not visible)

5. QRS duration (narrow / wide)

6. ST-segment/T-wave morphology (normal / not assessable / slight lateral ST depression / elevated / T-wave inverted)

7. QTc interval (normal / not assessable / prolonged QT interval / J wave visible after the QRS)

Report your responses in structured format with one parameter per line, providing only the selected response value for each parameter.
