# Supplementary material for: Artificial Intelligence for Biomedical Diagnostics: Diagnostic Accuracy and Reliability of Multimodal Large Language Models in Electrocardiogram Interpretation
Source: Life (Basel). 2026 Apr 16;16(4):681. doi: 10.3390/life16040681 (PMC13117897; doi:10.3390/life16040681)
Supplement: Supplementary file 1 [file life-16-00681-s001.zip › S1 Table.pdf]

Table S1. Diagnostic performance metrics for binary classification tasks based on majority vote predictions per ECG (n = 13).

Model	Rhythm Sens	Rhythm Spec	Rhythm PPV	Rhythm NPV	Rhythm Bal. Acc.	QRS Sens	QRS Spec	QRS PPV	QRS NPV	QRS Bal. Acc.
**ChatGPT-5.3**	0.0	100.0	n/a	84.6	50.0	50.0	100.0	100.0	91.7	75.0
**Gemini 3.1 Pro**	50.0	90.9	50.0	90.9	70.5	50.0	90.9	50.0	90.9	70.5
**Claude Opus 4.6**	100.0	63.6	33.3	100.0	81.8	50.0	63.6	20.0	87.5	56.8
**Grok 4.1**	0.0	81.8	0.0	81.8	40.9	50.0	90.9	50.0	90.9	70.5
**ERNIE 5.0**	0.0	90.9	0.0	83.3	45.5	0.0	100.0	n/a	84.6	50.0

Sens = sensitivity; Spec = specificity; PPV = positive predictive value; NPV = negative predictive value; Bal. Acc. = balanced accuracy, defined as (sensitivity + specificity) / 2; n/a = undefined due to zero counts in the denominator. For rhythm, the positive class is "irregular" (n = 2); for QRS, the positive class is "wide" (n = 2). All values should be interpreted descriptively given the small number of cases per class.
